# Changing the Face of Modern Medicine: Stem Cells & Gene Therapy, October 18–21, 2016, Florence, Italy

**DOI:** 10.1016/j.ebiom.2016.11.028

**Published:** 2016-11-25

**Authors:** Daniel W. Stuckey

## 1 Treating Age-related Macular Degeneration Using Human iPS Cells

Age-related macular degeneration (AMD) is a medical condition of the eye that results in blurred vision and/or a loss of central vision. The wet form of AMD – caused by abnormal blood vessel growth – is often treated using anti-VEGF medication. This approach can slow deterioration but is unable to treat the cause, or reverse the effects of, retinal pigment epithelium (RPE) atrophy. A regenerative medicine strategy currently being explored is the possibility that healthy RPE tissue could replace defective RPE. Masayo Takahashi from the RIKEN Center for Developmental Biology (Kobe, Japan) presented data on the creation and utilization of RPE cells from inducible pluripotent stem cells (iPSCs) to treat wet AMD. In 2014, Takahashi led a pilot study of the first transplant of human iPSC-derived tissue into a human being. Skin cells taken from a patient suffering from wet-AMD were converted into iPSCs, differentiated into RPE cells and transplanted back into the patient's eye. Importantly, transplanted cells were not rejected and resulted in the stabilization of visual acuity. However, using autologous cells is a costly option and is of limited use to patients with acute ocular diseases. To address these points, Takahashi has explored the therapeutic use of RPE cells from iPSCs derived from human leukocyte antigen (HLA)-matched homozygote donors. When tested in a monkey model, no rejection signs were observed in monkey iPSC-RPE allografts of MHC-matched animal models, whereas immune attacks around the graft were detected in the MHC-mismatched situation. Using an *in vitro* assay to assess the immune response in a quasi-human context, T cells were unable to respond to human iPSC-RPE cells derived from HLA homozygous donors. This encouraging result indicates that the allogeneic option is viable and should be tested in future clinical trials if the donor and recipient are HLA matched.

## 2 Remuscularization of Injured Hearts with Human ESC-Derived Cardiomyocytes

Myocardial infarction (MI) occurs when blood flow to the heart is prevented, usually as a result of a blood clot. This can cause damage to the heart muscle, resulting in death of cardiac tissue which is replaced with collagen scar tissue. Scar tissue is not contractile and does not possess the electrical conductivity of cardiac tissue, putting the patient at risk of heart failure and arrhythmias. Michael Laflamme (University Health Network, Toronto, Canada) presented preclinical data on the use of human embryonic stem cell-derived cardiomyocytes (hESC-CMs) to improve the function of infarcted hearts. Using a guinea pig model, when hESC-CM cells were grafted into injured hearts they were able to integrate into host heart muscle. Shared gap junctions between these tissues were observed along with an improved mechanical function in treated animals. To test host-graft electrical coupling, grafted tissue was labelled with a genetically-encoded calcium sensor. Host ECG could be correlated to calcium flux in donor tissue, demonstrating a certain degree of 1:1 host-graft coupling in damaged hearts. To determine if this approach could be translated into larger animal models where many more cells would be needed, a non-human primate model of myocardial ischemia-reperfusion was utilized. Macaques with MI were treated with 1 billion hESC-CMs, delivered to and around the infarct region. Autopsy showed significant remuscularization of the infarcted heart, with reciprocal gap junctions and integration of host vasculature into donor tissue. However, in contrast to small animal models, small non-fatal arrhythmias were observed in engrafted macaques. These results suggest that hESC-CM grafts are able to improve the function of infarcted hearts, but the challenge is to improve electrical properties of the graft to minimize arrhythmic complications. The next step is to refine this promising approach in a pig MI model which is more faithful to the human situation.

## 3 First Application of Gene-edited ‘Universal’ T Cells for Leukemia

Chemotherapy has been used with great success to treat patients with leukemia. Unfortunately, this approach is not always effective, especially when cancerous cells become refractory through resistance mechanisms. Until recently, palliative care has been the only option left for patients suffering from aggressive drug-resistant leukemia. However, developments in the field of immunology are revolutionizing the way hematological cancers are tackled. Waseem Qasim (University College, London) presented one such strategy that is being used with great success to treat CD19-positive B-cell Acute Lymphoblastic Leukemia (B-ALL). Essentially, T cells derived from healthy donors were molecularly tweaked using genome editing technology. These so-called UCART19 cells express chimeric antigen receptors (CARs) that target surface CD19 proteins found on leukemic cells, and kill them. CART19 cells were initially tested in two extremely sick children with relapsed B-ALL. Using a special license allowing application of unlicensed medical products, CART19 cells were administered. This was a landmark experiment in which gene-edited therapeutic T cells were being tested in humans for the first time. Both patients responded remarkably well to the treatment, showing robust abolition of leukemic cells. This pioneering study informed the dosing and schedule of a Phase 1 trial to evaluate the safety and ability to induce molecular remission in pediatric patients with relapsed/refractory CD19-positive B-ALL, taking place at Great Ormond Street hospital (NCT02808442). This trial is currently recruiting participants and hopes to prove the effectiveness of UCART19 cells in larger groups of patients. Similar studies are needed to test if this therapeutic approach can be expanded to treat other aggressive forms of chemotherapy-resistant cancer.

## 4 Generating a Kidney From Human Pluripotent Stem Cells: Where to From here?

In a plenary session dedicated to new technologies, Melissa H. Little from the University of Melbourne (Melbourne, Australia), discussed the generation of kidney organoids from hPSCs and posed the question: where to from here? Approximately 10% of Australian's suffer from chronic kidney disease, driving the need to better model diseases in the laboratory to dissect etiological mechanisms, screen therapeutic agents and explore the possibility of regenerative applications. Little presented a protocol to differentiate hPSCs into all four kidney progenitor populations (nephron, ureteric epithelial, renal interstitial and endothelial), that could be further directed to form self-organized kidney organoids. Detailed analysis of these 3D organoids identified at least nine different kidney cell types, and transcriptional profiling indicated similarity to that of a first trimester fetal kidney. Functional testing demonstrated that these kidney organoids exhibit some of the actions of an intact kidney, including formation of a brush border within the proximal tubules that can absorb nutrients. These structures also express multiple drug transporters that respond to nephrotoxins, making them appropriate for drug screening assays. However, fundamental processes such as blood filtration and urine production are not present and introducing additional steps or bioengineering methods to refine the protocol might expand organoid functionality. Nevertheless, creating kidney organoids from patient-derived iPSCs provides a means to understand and validate novel mutations. One third of kidney diseases are inherited, so recapitulating renal kidney disease in the dish will enable the development of mutation-specific therapies and even correct the mutation using gene editing technologies. Perhaps in the future, corrected kidney organoids could be transplanted back into the patient to replace defective kidney tissue. Organoids recapitulating many different body parts have now been created, providing an *in vitro* platform to model disease and test therapeutic interventions.

## 5 Genome Engineering Using CRISPR-Cas: Prospects and Challenges

Feng Zhang from The Broad Institute MIT (Cambridge, USA) gave an inspiring talk on the prospects and challenges of genome engineering. The presentation covered two main topics. First, strategies to improve the specificity of genome editing were discussed. A propensity for RNA-guided nucleases to cut at non-specific sites, resulting in unintended mutagenesis, has hampered the use of CRISPR-Cas in situations requiring high levels of specificity. Zhang's approach to tackle this issue was to use structure–guided protein engineering of *Streptococcus pyogenes* Cas9 (SpCas9), to abolish off-target effects while preserving on-target activity. The crystal structure of SpCas9:DNA indicated that positively charged amino acids were found in the cleft of SpCas9 that holds the negatively charged non-target DNA strand. These residues could be systematically substituted for neutral amino acids to reduce this binding and, in theory, necessitate more stringent base pairing between the RNA guide and target DNA strand to improved specificity. Indeed, a number of mutants were identified that were able to reduce off-target activity by at least 10-fold compared to wildtype SpCas9, while maintaining on-target cleavage activity. The second topic discussed was how bacterial diversity could be explored to identify new CRISPR-Cas systems. To date, only two Class 2 nucleases – those with a single effector subunit – have been described, Cas9 and Cpf1. This runs counter to the extreme diversity shown by microbes that are likely to have evolved additional Class 2 variants. Using a computational prediction approach across microbial genome sequences, new candidate Class 2 CRISPR-Cas9 loci were identified. A total of 53 loci were discovered, that were divided into three distinct groups based on the nature of their effector proteins, denoted C2c1–3 (Class 2 candidate 1–3). The functionality of these Class 2 variants are currently being tested, and will perhaps offer novel mechanisms of action to expand the CRISPR-Cas toolkit.

## 6 Use of Adipose Stem Cells to Treat Inflammatory Diseases

In a session dedicated to MSC Gene & Cell Therapy, Wilfried Daelemans from TiGenix (Leuven, Belgium), presented data on the use of expanded allogeneic Adipose-derived Mesenchymal Stem Cells (ASCs) to treat inflammatory disorders. The rationale for using ASCs is that they possess anti-inflammatory and immunomodulatory potential, whilst being well tolerated. ASCs are separated from liposuction-derived adipose fat, characterized, expanded and stored in a cell bank until required. The use of ASCs in a variety of preclinical mouse models of inflammation were presented. Administration of ASCs resulted in decreasing the severity of established arthritis in a collagen-induced mouse model for rheumatoid arthritis, primarily by restoring the Treg compartment in treated animals. Similarly encouraging results were seen in a dextran sulfate sodium-induced model of colitis, and in both lipopolysaccharide and cecal ligation and puncture models of sepsis ASC-treated mice showed decreased mortality compared to non-treated controls. To illustrate the success of this approach in humans, results from a recently completed clinical trial were highlighted. This Phase 3 randomized, multicenter trial aimed to evaluate the safety and efficacy of allogeneic ASCs (Cx601) for treatment of treatment-refractory complex perianal fistulas in Crohn's disease patients (NCT01541579). The primary endpoint was met with half of the patients treated with a single injection of Cx601 achieving remission at week 24. Cx601 was well tolerated in the study population and no immune reaction or specific treatment emergent adverse events were detected. Follow-up analysis at 52 weeks confirmed the sustained efficacy and safety profile of Cx601. As a result of these encouraging results, TiGenix has submitted a Marketing Authorization Application to the EMA and is preparing to develop Cx601 in the United States after having reached an agreement with the FDA. Other ASC products are in the clinical pipeline at TiGenix to treat a range of inflammatory diseases that are treatment-refractory, underscoring the clinical utility of ASCs as a therapeutic entity.

